# Graph and Hypergraph Theories Applied to Dynamic Protein–Protein Interaction Network Analysis, and Deep-Learning Frameworks for Protein Complex Network Prediction

**DOI:** 10.3390/ijms27114750

**Published:** 2026-05-25

**Authors:** Kai-Yu Chan, Tatsuo Yamaguchi, Yoshihiro Izumiya, Yen-Wei Chu, Tadashi Watanabe

**Affiliations:** 1 Graduate Institute of Genomics and Bioinformatics, National Chung-Hsing University, Taichung 40227, Taiwan; g113019007@smail.nchu.edu.tw; 2 Laboratory of Bioinformatics, Artificial Intelligence Center for Health and Biomedical Research, National Institutes of Biomedical Innovation, Health and Nutrition, Settsu 566-0002, Osaka, Japan; yomiyama1998@nibn.go.jp; 3 Department of Dermatology, School of Medicine, University of California Davis, Sacramento, CA 95817, USA; yizumiya@health.ucdavis.edu; 4 Doctoral Program in Medical Biotechnology, National Chung Hsing University, Taichung 40227, Taiwan; 5 Institute of Molecular Biology, National Chung Hsing University, Taichung 40227, Taiwan; 6 Smart Sustainable New Agriculture Research Center (SMARTer), National Chung Hsing University, Taichung 40227, Taiwan; 7 Department of Virology, Graduate School of Medicine, University of the Ryukyus, Ginowan 901-2720, Okinawa, Japan; 8 Molecular Microbiology Group, Department of Infectious Diseases, Tropical Biosphere Research Center, University of the Ryukyus, Nishihara 903-0213, Okinawa, Japan

**Keywords:** protein–protein interaction networks (PPINs), protein complex network (PCN), centralities, hypergraph, machine- and deep-learning

## Abstract

Protein interactions form large-scale networks known as protein–protein interaction networks (PPINs) or protein complex networks (PCNs). Extracting meaningful structural frameworks from these molecular relationships through mathematical modeling enables a deeper understanding of biological processes. Although static protein network models have provided valuable insights into the organization of PPINs, they are limited in their ability to capture the dynamic and cooperative nature of protein complexes. This review begins by introducing fundamental concepts in graph and hypergraph theory, with an emphasis on centrality measures. We then discuss the evolution of PPIN analysis from static representations to dynamic graph- and hypergraph-based frameworks. Specifically, we review dynamic PPINs and the challenges associated with their interpolation, dynamic centrality measures, and network models capable of representing multi-node relationships that have been applied to PPINs. Finally, we highlight recent advances in machine learning and deep learning approaches that integrate interaction data with functional annotations, sequence information, and cellular context to predict novel interactions and reconstruct transient protein complexes. Taken together, dynamic PPIN modeling combined with experimental validation provides an integrated framework for understanding coordinated protein functions in cellular processes and across biological systems as well as supporting drug development.

## 1. Introduction

Proteins are long-chain molecules composed of amino acids and serve as the fundamental molecules of life. The sequence and composition of amino acids in a protein determine its diversity and primary function. Protein–protein interactions (PPIs) mediate biological activities in living cells. In intracellular processes, proteins interact with and respond to the cellular environment, which is influenced by post-translational modifications [1], local protein concentrations, often enhanced through phase separation [2], and the presence of specific protein partners that induce conformational changes known as allosteric regulation [3]. These factors collectively coordinate biological cascades and signaling networks, ultimately determining cellular outcomes [1,2,3]. The regulation of PPIs is essential for numerous biological processes and cellular responses. Therefore, the prediction and identification of PPINs are critically important and have long represented a major challenge in the life sciences [4,5,6,7,8,9,10,11]. Recent advances in proteomics technologies have led to a substantial increase in available PPI data, which continue to grow exponentially. Such information is publicly accessible through PPI databases, including BioGRID [12] and STRING [13]. Furthermore, PPI data are integrated with existing information resources that provide information on protein function, interaction specificity, and structural features. These comprehensive protein information resources have significantly contributed to progress in the field. A summary of representative experimental methods for PPI detection is provided in Table 1.

The increasing volume of PPI datasets generated by high-throughput technologies facilitates biochemical studies and contributes to elucidating the functions of proteins of interest. However, current approaches primarily represent static PPINs derived from previously identified interactions through affinity capture-MS and/or yeast two-hybrid studies. In reality, PPINs within cells are highly dynamic and continuously change in response to external environmental conditions. Consequently, recent research has shifted toward the development of algorithms that represent dynamic PPINs [14] and predict changes in protein complexes regulated by the addition (e.g., overexpression) or removal (e.g., genetic deletion) of specific proteins.

Current platforms for dynamic PPIN construction are typically based on static PPINs, incorporating protein activity probabilities inferred from gene expression profiles. Gene expression levels are quantified at each time point, and correlation coefficients are calculated and visualized. The integration of transcriptomic datasets is expected to improve the accuracy of predicted protein complex networks. Although this approach can describe correlations between expression levels and the probability that a protein is present within a complex, thereby capturing aspects of protein complex dynamics, it cannot account for PPIs that have not yet been identified. Numerous prototype frameworks for dynamic PPIN analysis have been developed using various algorithms [15,16]. However, existing analytical tools have not yet resolved the issue of frame rate limitations, which primarily arise from handling and visualizing PPINs as distinct time points. This leads to sequential variability due to the large number of proteins and PPIs involved, without preserving temporal continuity.

PPIN modeling is conventionally represented as a graph, in which nodes correspond to proteins and edges represent PPIs. Graphs are highly useful network models for illustrating relationships between pairs of entities. However, a fundamental limitation of graph representations is their inability to distinguish whether proteins A, B, and C form a single protein complex, three distinct pairwise interactions (A–B, B–C, and C–A), or a simultaneous interaction among all three proteins. This limitation is particularly important for understanding the regulatory mechanisms of large protein complexes, such as transcriptional coactivators, corepressors, and epigenetic remodeling complexes. Notable examples include the RNA polymerase II complex, which is essential for transcription and consists of at least 12 distinct proteins [17]. Another example is the SWI/SNF epigenetic remodeling complex, which regulates transcription by modulating nucleosome positioning at specific genomic regions. The SWI/SNF complex can assemble into multiple distinct configurations comprising 8 or more proteins through the exchange of its constituent subunits [18]. Genomic mutations in SWI/SNF components are frequently observed in cancer, and understanding how these mutations affect complex functionality is critical for elucidating disease mechanisms. Therefore, a novel PPIN modeling framework that represents a protein complex as a unified entity and predicts the effects of single-protein perturbations (e.g., deletion) would be of significant value. Predicting potential interacting protein partners requires assigning quantitative values to each protein node, which can be achieved using centrality measures. Centrality quantifies the relative importance of nodes within a graph and has been widely applied to PPINs to characterize protein complex organization.

**Figure 1:**
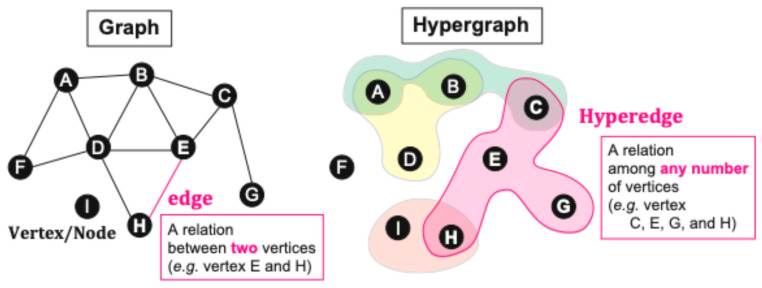
Conceptual comparison between graphs and hypergraphs. Hypergraphs extend graphs by capturing multi node relationships beyond simple pairwise interactions. (**Left**) A graph represents pairwise relationships, where an edge connects exactly two nodes (e.g., E–H). (**Right**) In contrast, a hypergraph uses hyperedges that connect multiple nodes simultaneously (e.g., C, E, G, and H), enabling the representation of higher order interactions such as protein complexes.

Furthermore, the limitations inherent in using graphs as representations of PPIs can be addressed by applying algorithms based on hypergraphs, a network model in which edges (termed hyperedges) can connect more than two vertices (Figure 1). Indeed, it has been proposed that protein complexes should be represented as hyperedges within a hypergraph [19].

Several excellent review articles have previously summarized PPIN analyses [20,21,22,23]. In this review, we present current perspectives on PPIN research and introduce novel mathematical concepts aimed at developing new analytical frameworks for studying PPIN dynamics. We also discuss the limitations of existing analytical tools.

This review focuses on the following topics:

- Dynamic PPINs and their associated interpolation challenges,
- Centrality measures, with particular emphasis on dynamic-specific approaches, and
- Network models capable of representing multi-node relationships that have been applied or are potentially applicable to PPINs.

## 2. Dynamic PPIN

**Figure 2:**
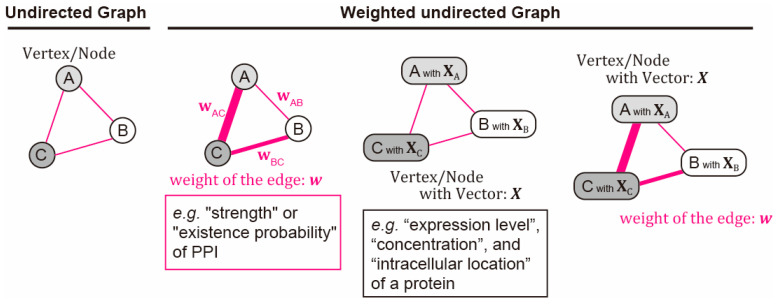
Concepts of undirected graph and weighted undirected graph on the PPIN. Weighted graphs and node features enhance PPIN representations by integrating quantitative interaction strengths and biological context beyond simple connectivity. (**Left**) An undirected graph represents PPINs in which nodes correspond to proteins and edges indicate pairwise interactions without directionality. (**Middle-Left**) A weighted undirected graph extends this framework by assigning a weight (w) to each edge, reflecting properties such as interaction strength or confidence. (**Middle-Right**) Nodes can be associated with feature vectors (X) that incorporate biological attributes such as expression level, concentration, or subcellular localization. (**Right**) The combination of weighted edges and node feature vectors enables more informative network modeling.

A static PPIN is defined as an undirected graph $G=\left(V,E\right)$ consisting of a vertex set V with proteins as vertices and an edge set E with interactions between two proteins as edges (Figure 2). The edge set E is a subset of the entire two-point subset {{u,v}⊂V∣u,v∈V}. We can also consider a weighted undirected graph $G=\left(V,E,w\right)$ that considers the weights of the edges w:E→R. The edge weight can be used, for example, to express the “strength” or “existence probability” of an interaction (Figure 2).

Similarly, nodes can be weighted, but in more detail, they can also be assigned a vector $X_{v}\in R^{d}\;\left(v\in V\right)$ of higher dimensions. This vector is referred to as a node feature or attribute. In protein analysis, node features enable the incorporation of data such as expression levels (i.e., the estimated abundance of a protein), concentrations, intracellular localization, and presence probabilities (Figure 2). Furthermore, community detection methods that integrate node features have been developed and may prove highly effective for PPIN applications, enabling the detection of cellular protein communities such as functional complexes or signaling cascades.

**Figure 3:**
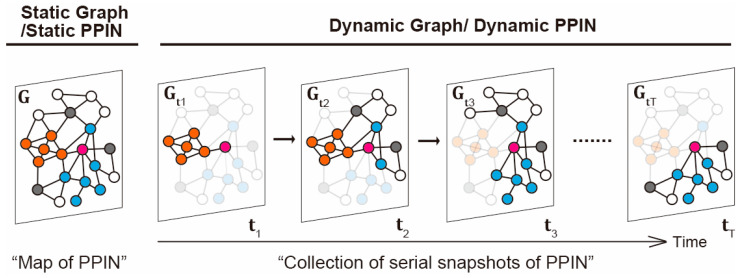
Static and dynamic representations of PPINs. While static PPINs provide a simplified overview of network structure, dynamic PPINs better reflect the time-dependent and condition-specific nature of protein interactions. (**Left**) A static PPIN represents PPIs as a single, time-aggregated network, providing a snapshot of connectivity without accounting for temporal changes. (**Right**) In contrast, a dynamic PPIN models the network as a series of time-resolved snapshots (t_1_, t_2_, t_3_, …, t_T_), where nodes and edges can change over time. This framework captures the temporal variability of interactions driven by biological processes such as cell cycle progression, signaling events, or environmental responses.

A dynamic PPIN is defined as a collection $\left\{G_{t_{1}},\ldots ,G_{t_{T}}\right\}$ of static PPINs $G_{t_{i}}=\left(V_{t_{i}},E_{t_{i}}\right)$ (i=1,…,T) as a series of snapshots (Figure 3). Here, $t_{i}$ can be given as a timestamp or condition. Dynamic networks are also known as temporal networks.

The main approaches currently used to construct dynamic PPINs can be broadly categorized as follows:

- Node-centric methods, which focus on node dynamics: These approaches are based on the dynamics of protein expression levels, governed by mRNA synthesis and protein degradation, as well as the spatiotemporal behavior of proteins determined by their intracellular localization.
- Edge-centric methods, which focus on edge dynamics: These approaches focus on changes in PPIs and their correlations, including variations in binding affinity and interaction modes, which are driven by both intrinsic protein properties and specific cellular conditions.

It is well-established that proteins can be expressed either transiently or stably, and that many undergo translocation to, or localization within, specific cellular compartments. Methods for modeling node and edge dynamics are based on these fundamental characteristics of cellular proteins. To capture these properties and develop more robust dynamic PPIN models, it is essential to integrate multiple datasets and experimental insights.

Androulakis I.P. et al. reviewed the qualitative characteristics of high-throughput data collection approaches, such as time-series gene expression, along with the current landscape of computational methods. They also highlighted key challenges in analyzing complex datasets, including the integration of knowledge-based clustering using Gene Ontology (GO) information [24]. Furthermore, Przytycka T.M. et al. provided a comprehensive overview of the transition from static to dynamic interactome analysis in computational biology. They emphasized the importance of incorporating diverse dynamic data, such as transcriptomic profiles and phenotypic effects of genetic perturbations (e.g., gene silencing or knockout), into proteomic frameworks [25].

### 2.1. Development of Dynamic PPIN Modeling

By integrating gene expression data and functional annotations with a static PPIN map, Lin C.-C. et al. developed a network-based comparative analysis approach to identify dynamic functional modules (i.e., protein clusters) across distinct biological states [26]. Specifically, gene expression profiles from dilated and non-dilated cardiomyopathy were used to construct co-expression-based PPINs by extracting correlated interactions from the static PPIN map. These networks were subsequently analyzed for functional classification using GO annotations, followed by a targeted module selection process. Ultimately, their analysis revealed that two functional PPI modules, muscle contraction and organ morphogenesis, are closely associated with heart failure.

Li M. et al. proposed a framework that integrates time-series gene expression data with PPI data to more effectively identify protein complexes and functional modules compared with their previous work [27,28]. Their study utilized multiple time-series transcriptomic profiles of S. cerevisiae throughout the cell cycle. The proposed algorithm improves the ability to distinguish functional modules from protein complexes and suggests that certain functional modules may span multiple complexes. In this study, a three-sigma method was applied to identify the active time points (activation timing) of each protein during the cell cycle [28]. Rather than relying solely on gene expression levels, this method determines an activation threshold for each protein based on the specific characteristics of its gene expression profile. This approach is based on the concept that protein activity is determined not only by its current state and local environment (including PPIs) but also by its cellular residence time, which is an indicator derived from gene expression and a critical factor in sustaining activity. In their framework, a dynamic PPIN can be constructed by integrating protein activation time-point information derived from this method with a static PPIN.

Ou-Yang L. et al. constructed dynamic PPINs by integrating static PPIN data with time-series gene expression data. In their framework, stable interactions consist of globally co-expressed proteins, whereas transient interactions involve proteins that are active at specific time points identified from time-series analysis [29]. They also proposed the time smooth overlapping complex detection (TS-OCD) model to predict temporal protein complexes from the resulting dynamic PPINs. This model captures the temporal smoothness of the network between consecutive time points and enables the identification of overlapping protein complexes at each time point.

Zhang Y. et al. constructed dynamic PPINs by integrating gene expression data into a static PPIN, introducing the concept of an active probability for both proteins and their interactions [30]. Furthermore, they developed a method for predicting protein complexes based on the core–attachment structural features within dynamic PPINs. By effectively leveraging both temporal activation probabilities and network topological information, they demonstrated a significant improvement in prediction accuracy compared with existing methods.

Sabzinezhad A. et al. constructed a dynamic PPIN by integrating tandem affinity purification (TAP) data, which are direct biological PPI datasets, with GO information to reduce noise in PPINs [31]. Additionally, they generated dynamic subnetworks by applying biclustering (the simultaneous clustering of two dimensions, i.e., a matrix of proteins and time points) to gene expression data, followed by the application of a memetic algorithm (an advanced form of genetic algorithm) to each bicluster. These subnetworks served as the basis for detecting protein complexes. This approach, known as the dynamic method, detects protein complexes from the TAP-aware weighted PPIN (DPCT) method, and was proposed as a novel and effective framework for the dynamic identification of protein complexes.

### 2.2. Dynamic Models Beyond PPIN: Gene Expression or Regulation

Several studies have explored dynamic models of gene expression and regulation, which are indirectly related to PPINs. Conventional dynamic gene expression models often assume that the network structure remains stable across time points while evolving only gradually toward a target structure. However, gene expression can change drastically in response to stimuli, leading to abrupt network transitions at specific time points. Consequently, the inherent instability of network structures at these critical time points may undermine the reliability of standard dynamic modeling approaches.

To address this issue, Yoshida R. et al. proposed a dynamic linear model incorporating Markov switching to estimate time-dependent gene network structures from time-series expression data [32]. Their method can predict the specific time points at which network structures undergo transitions. Similarly, to better understand gene regulatory networks in dynamic environments, Rao A. et al. introduced the regime state-space model (regime-SSM) [33]. This approach employs clustering based on basal dynamics, followed by the application of a state-space model to each cluster for system identification. This framework enables the inference and representation of time-varying networks that reflect diverse cellular states. Furthermore, Lèbre S. et al. developed the auto-regressive time-varying (ARTIVA) model, a statistical framework designed to infer temporally varying gene regulatory networks [34]. ARTIVA enables the reconstruction of temporal sequences of regulatory interactions for genes involved in specific biological processes, such as development or stress responses, by jointly inferring network topology and its temporal evolution.

While no single model is universally optimal, each offers distinct strengths and limitations. Integrating these diverse mathematical approaches can provide a more accurate representation of the complex mechanisms underlying gene expression and regulation in cells.

### 2.3. Approaches for Functional Module Detection in Dynamic PPINs

Several analytical methods have been proposed to identify functional modules in dynamic PPINs. Analyzing microarray data obtained under various conditions from the public yeast gene expression database (Saccharomyces Genome Database; SGD), together with known yeast PPINs, Komurov K. et al. identified differences in the behavior and properties of dynamic and static functional modules (i.e., clusters of proteins) by examining their local network positions [35]. Stably expressed proteins are associated with static functional modules, whereas dynamically regulated proteins are associated with dynamic modules. Based on these findings, the authors also suggested the potential for improving dynamic modularity models of PPINs. Ou-Yang L. et al. integrated multiple clustering results to construct a protein consensus matrix. To generate a co-complex similarity network, this matrix was combined with co-complex score matrices derived from tandem affinity purification/mass spectrometry (TAP/MS) data [36]. Based on the resulting network, a two-layer integrated complex detection (TINCD) model was developed as a novel method for detecting protein complexes. These studies highlight the importance of integrating heterogeneous data and dynamic information for accurate functional module detection in PPINs.

### 2.4. Network Interpolation and Extrapolation in Dynamic PPINs

Dynamic PPINs constructed from time-course gene expression datasets represent an important approach for understanding temporal network behavior. However, gene expression data available in public databases often lack sufficient temporal resolution to capture dynamics occurring over short time scales. Therefore, it is crucial to determine how finely time steps should be discretized and how consecutive time points should be connected.

Tasks involving the prediction of networks are referred to as network inference tasks, and the interpolation problem in dynamic networks falls within this category. Network interpolation refers to the prediction of an intermediate network between two static networks, where each static state is treated as a “snapshot”. For a dynamic graph $\left\{G_{t_{1}},\;G_{t_{2}},\;\ldots ,\;G_{t_{T}}\right\}$ and a set of time steps $t_{1}<t_{2}<\cdots <t_{T},$ the interpolation problem can be defined as predicting an intermediate network $G_{t}$ for $t_{i}<t<t_{i+1}$. In other words, interpolation aims to model the transition of the network structure between the “start” and “end” states of the network at consecutive time points.

Similarly, extrapolation refers to the prediction of future networks, i.e., estimating $G_{t}$ for $t>t_{T}$. This task is generally more challenging than interpolation because the final network structure is unknown. While short-term predictions may be feasible, forecasting distant future states is considerably more difficult. Small estimation errors accumulate over time, leading to increasing uncertainty, analogous to the uncertainty inherent in long-range weather forecasting.

Several mathematical approaches have been proposed to address interpolation problems based on snapshots. Elzen S.v.d. et al. treated each network snapshot as a point in a coordinate space and visualized network evolution by overlaying two representations: one showing the network structure and the other illustrating its temporal progression [37]. This approach enables the identification of network stability, recurring patterns, and anomalous behavior.

Bakker C. et al. applied interpolation and averaging between snapshots using a Riemannian geometry-based framework to the problem of dynamic community detection [38]. They demonstrated that this approach is more suitable than simple linear interpolation. Although their primary aim was to improve the adaptability and efficiency of community detection, their work also provides important insights into interpolation methods for dynamic networks.

### 2.5. Graph Representation Learning

As an alternative approach to addressing the interpolation problem, graph representation learning can be applied. Graph representation learning is a general framework that embeds nodes of a graph into a coordinate space, thereby enabling the application of machine learning and other computational techniques. Hamilton W.L. et al., in their review, describe several representation learning methods for static graphs [39]. Readers may also refer to a comprehensive review by Kazemi S.M. et al., which summarizes recent advances in representation learning for dynamic graphs [40]. Notably, the authors highlight progress in the field of knowledge graphs (KGs) as a key example. A KG is a graph in which edges are annotated with explicit relation types (e.g., interaction, phosphorylation, or degradation), making it a suitable framework for capturing the relational diversity of PPIs in network analysis.

Graph neural networks (GNNs) are neural network models designed to operate on graph-structured data and have been widely adopted for graph representation learning. Although GNNs have achieved considerable success on static graphs, their performance on dynamic graphs has remained limited. To address this limitation, You J. et al. proposed the ROLAND framework, which enables the adaptation of GNNs from static to dynamic graph settings through a hierarchical state update mechanism [41]. Furthermore, dynamic graph neural networks (DGNNs) have emerged as an important and active area of research. A dedicated review by Yang L. et al. provides a comprehensive survey of DGNN architectures, training strategies, and applications [42].

A comparison of representative static and dynamic PPIN modeling approaches, including their input data, analytical tasks, applications, and limitations, is summarized in Table 2.

## 3. Centrality Measures

The node (or edge) centrality, or simply centrality, is a measure of the importance of a node. Mathematically, this is expressed as a function φ:V→R (or φ:E→R) that assigns a real number to a node (or edge). Numerous definitions of centrality exist, but which centrality to focus on depends on the properties of the node or edge of interest. Many centrality measures are determined by the topological and geometric properties around a node.

In PPINs, centrality is treated as an indicator used to address the question “Which proteins are important and why?”. Many studies have been devoted to revealing the relationship between certain centrality measures in PPINs and the essentiality of proteins. For example, essentiality could refer to cellular lethality when the protein is removed from a living cell. Research addressing this concept was first conducted by Jeong H. et al. using yeast PPINs [43]. They found a strong correlation between degree centrality and lethality caused by protein deletion. This relationship is well-known as the centrality–lethality rule. Furthermore, centrality has been shown to be associated with biological properties beyond essentiality. For example, the degree centrality (DC) of proteins in PPINs has been reported to be negatively correlated with their evolutionary rate, measured as the rate of amino acid substitutions [44]. This suggests that proteins with many interaction partners tend to evolve more slowly.

Some centrality measures are defined for dynamic networks. The importance of such centralities is expected to increase in future research. In this section, we primarily list some of the popular static centralities. Then, we describe centralities that are specific to dynamic networks.

The centralities explained here are mainly classified into the following two categories in PPINs:

- Local topological characteristic-based centralities in static/dynamic PPINs,
- Path- and walk-based centralities in static/dynamic PPINs.

Local topological characteristic-based centralities describe the structural properties of a node within its immediate neighborhood, such as the number and arrangement of directly connected interactions. In contrast, path- and walk-based centralities characterize the role of nodes in the global network structure, particularly in terms of their involvement in information transmission and connectivity between distant nodes.

### 3.1. Local Topological Characteristic-Based Centralities in Static PPINs

As discussed above, PPINs provide a fundamental framework for understanding cellular processes in which proteins interact to form complex functional systems. To quantitatively evaluate the importance of individual proteins in such networks, centrality measures have been widely used. In static PPINs, interactions are represented as fixed relationships, and local topological characteristics, such as node connectivity, serve as fundamental measures for evaluating node importance. These measures can also be applied to dynamic PPINs by analyzing individual snapshots; however, they do not fully capture temporal variations. In this section, we introduce representative centrality measures based on local topological characteristics in the context of static PPINs.

In an undirected graph $G=\left(V,E\right)$, the number of edges connected to a node v is called the degree centrality (DC) of v and is written as $d_{v}$. In PPINs, a protein with large degree centralities interacts with many other proteins, and such a protein is called a hub protein. Jeong H. et al. found that the majority of proteins had small degree centralities. On the other hand, a small number of proteins had a large degree centrality [43]. This implies that the degree distribution of PPINs is “scale-free”. In addition, as above-mentioned, they compared degree centrality with the lethal phenotype of protein removal and found a strong correlation between degree centrality and the lethal phenotype. Tang X. et al. used Pearson’s correlation coefficient (PCC) to bridge the gap between PPI and gene expression data [45]. Based on PCC and the edge clustering coefficient (ECC), weighted degree centrality (WDC) was introduced to improve the reliability of the prediction of essential proteins.

For a graph G, the adjacency matrix $A=(A_{uv})\in R^{n\times n}$ is defined by

$$A_{uv}=\left\{\begin{array}{ll} 1 & \mathrm{if}\;\;\left\{u,v\right\}\in E,\;\\ 0 & \mathrm{if}\;\;\left\{u,v\right\}\notin E. \end{array}\right.$$

**Figure 4:**
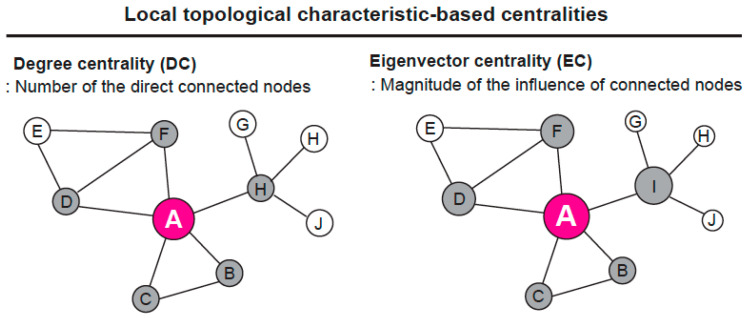
Representative local topological characteristic based centralities in PPINs. Local centrality measures quantify the importance of a node based on its immediate network neighborhood. In both panels, node A represents the central node whose importance is being evaluated. (**Left**) Degree centrality (DC) is defined as the number of directly connected nodes (gray-circles: e.g., B, C, D, F, H), reflecting local connectivity. (**Right**) Eigenvector centrality (EC) extends this concept by considering not only the number of connections but also the influence of neighboring nodes (indicated by the size of each gray-circles: e.g., I > F = D > B = C), assigning higher scores to nodes connected to highly influential nodes. These measures are typically applied to static PPINs but can be extended to dynamic PPINs. Different centrality measures capture distinct aspects of node importance. In summary, DC reflects local connectivity, whereas EC captures influence within the broader network context.

Eigenvector centrality (EC), denoted by $\mathrm{EC}\left(v\right)$, is defined by the entry $x_{v}$ of an eigenvector x of A. Typically, the eigenvectors for the largest real eigenvalue are used. EC was introduced by Bonacich P. et al. [46]. Because

$$\mathrm{EC}\left(v\right)=x_{v}=\frac{1}{\lambda }\sum_{u\in V}A_{vu}x_{u}$$

holds for a real eigenvalue λ, EC can be considered as an indicator of the magnitude of the influence of neighboring nodes. More generally, the type of centrality derived from the eigenvectors of a positive entry matrix (called a centrality matrix) is sometimes called eigenvector centrality, including α-centrality [47], PageRank centrality, Katz centrality, hub and authority scores (also known as HITS, Hyperlink-Induced Topic Search), and many others. Figure 4 summarizes the definitions of the representative local topological characteristic-based centralities, DC and EC.

### 3.2. Path- and Walk-Based Centralities in Static PPINs

In addition to local connectivity, the global structure of the network also plays an important role in determining node importance. As discussed in Section 1, biological functions often arise from coordinated interactions among multiple proteins, which can be represented as pathways and patterns of information flow in PPINs. In static networks, path- and walk-based centralities are measures that evaluate how efficiently a node can reach other nodes or mediate interactions within the network. In this section, we review representative path- and walk-based centralities in static PPINs.

For nodes u, v∈V, the distance between u and v is the length of the shortest path from u to v. A path, defined as a route from u to v, is a sequence of edges and nodes. Closeness centrality (CC), denoted by $\mathrm{CC}\left(v\right)$, of node v is defined by the inverse of the average of the distances:

$$\mathrm{CC}\left(v\right)=\frac{1}{\frac{1}{\left|V\right|-1}\sum_{u\neq v}d\left(u,v\right)}.$$

On average, CC is larger for nodes with shorter distances to all nodes.

For nodes u,w∈V, let $\sigma_{uw}$ be the number of the shortest paths between u and w. On the other hand, let $\sigma_{uw}(v)$ for a node v be the number of shortest paths between u and w passing through v. Betweenness centrality (BC), denoted by $\mathrm{BC}\left(v\right)$, is defined by

$$\mathrm{BC}\left(v\right)=\sum_{u,w\neq v}\frac{i_{uw}\left(v\right)}{i_{uw}}.$$

This value indicates whether a node v in the graph G is a relay point between two other nodes. Joy M.P. et al. analyzed BC in yeast-PPINs and found that proteins with high BC and small DC were abundant [48]. This finding may indicate that these proteins act as important links between protein complexes and contribute to the emergence of a modular structure in the PPIN. Proteins with large BC, key connectors between interacting modules, are called bottleneck proteins [49]. Bottleneck proteins have been found to be less co-expressed with their neighbors than non-bottleneck proteins. The study suggests that bottleneck protein expression is critical for driving changes in the connection between biological modules and strongly affects the network topology. BC is often cited as a centrality that represents a bottleneck, but its definition implies that a node with a large BC is also a node that is part of a “highway”, connecting modules via the shortest paths.

In contrast, Pržulj N. et al. defined bottleneck centrality (BNC) as a more intuitive measure of bottleneck properties as follows [50]. For a graph G and node v, a tree Tv is obtained by taking the shortest paths from v to the other nodes [50]. For this Tv, the weight of a node u∈V is defined by the number of paths below u. This corresponds to the number of shortest paths from v that pass through u. A node u is called a bottleneck if its weight is larger than |V|/4. Therefore, “BNC” (v) is defined as the number of bottleneck nodes for v. The nodes that occur frequently as bottlenecks are defined as “important nodes” [50]. It was found that the proteins corresponding to the “important nodes” were inviable and were often structural proteins. From the study, they concluded that such proteins are more likely to be used to build and support structures than to transduce intracellular signals.

For node v, $\mu_{k}(v)$ is defined by the number of closed walks that return to v in k steps. Then, subgraph centrality (SC), denoted by $SC\left(v\right)$, is defined by

$$\mathrm{SC}\left(v\right)=\sum_{k=1}^{\infty }\frac{\mu_{k}(v)}{k!}.$$

**Figure 5:**
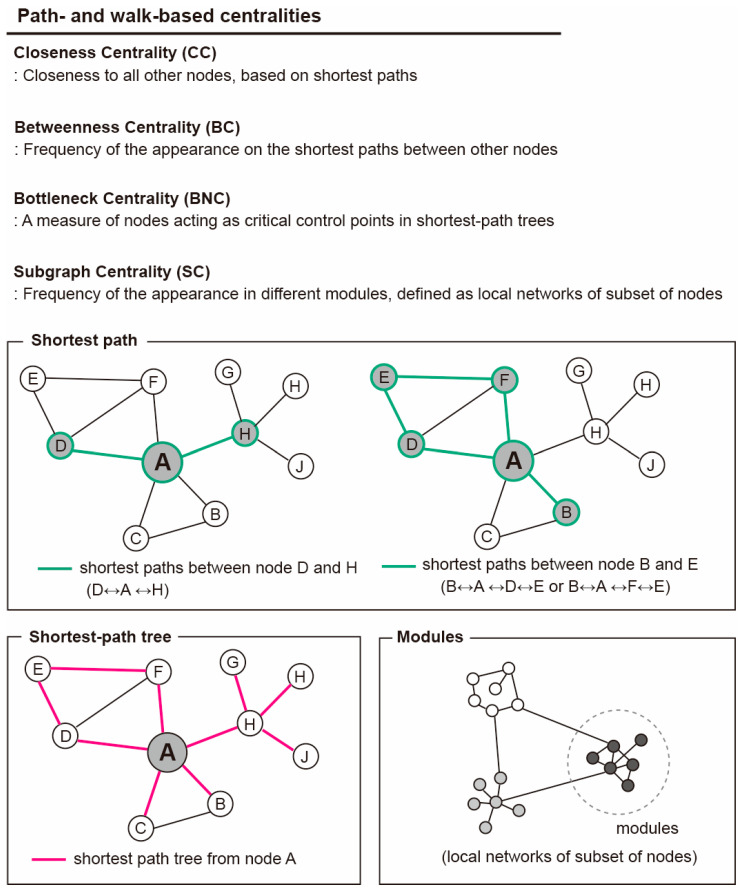
Representative path- and walk-based centralities and related network concepts. Unlike local centralities, path and walk based measures shed light on nodes that control information flow, connectivity, and modular structure within PPINs. Path- and walk-based centrality measures evaluate node importance based on global network structure. Closeness centrality (CC) quantifies how close a node is to all other nodes based on shortest path distances. Betweenness centrality (BC) measures how frequently a node appears on shortest paths between other nodes, reflecting its role as a mediator. Bottleneck centrality (BNC) identifies nodes that act as critical control points in shortest path trees. Subgraph centrality (SC) captures the frequency of a node’s participation in local subgraphs or modules. The lower panels illustrate key concepts underlying these measures, including shortest paths between node pairs, shortest path trees originating from a source node, and modules representing locally dense subnetworks.

This centrality, introduced by Estrada E. et al., provides a value corresponding to the frequency with which node v appears in different modules. This centrality was verified for yeast PPINs. As a result, the correlation coefficient of SC with essentiality is higher than for the other centralities such as DC, CC, BC, and EC [51]. While many centralities have been developed, the characteristics of the dataset and the purpose of the analysis determine the centrality to apply. Ashtiani M. et al. compared 27 centrality measures using yeast PPINs. After applying principal component analysis (PCA) and hierarchical clustering, they found that the most informative measures depended on the topology of the network [52]. In that meta-analysis, seven centrality measures showed a higher contribution than the others. Figure 5 summarizes the definition of the representative path- and walk-based centralities and illustrates the concepts of shortest path and related definitions.

### 3.3. Local Topological Characteristic-Based Centrality in Dynamic PPINs

Although the centrality measures discussed above are defined based on static network representations, PPINs are inherently dynamic systems, as described in Section 1. PPIs vary over time and under different biological conditions, and dynamic PPINs are often constructed using time-series gene expression data or condition-specific interactions. Under such circumstances, static centrality measures may fail to adequately capture temporal variations in node importance. Therefore, it is necessary to extend centrality measures to dynamic networks. In this section, we focus on dynamic centrality measures based on local topological characteristics in dynamic PPINs.

Han J-D. et al. investigated how ‘hubs’ contribute to the robustness and properties of dynamic PPIs [53]. In particular, they distinguished two types of hubs. One type is “party” hubs, which interact with most partners simultaneously. The other type is “date” hubs, which bind to different partners at different times or locations. Additionally, their analysis suggests that modular organization consists of date hubs connecting the biological processes or modules and the party hub networks within these processes and modules. Taylor I.W. et al. attempted to predict patient outcomes from the changes in the interactome structure [54]. Initially, they analyzed the hub proteins using gene expression datasets from about 80 human tissues. Through the analysis, the hubs were classified into two types: intermodular hub proteins exhibiting a low correlation of co-expression with interactants, and intramodular hub proteins exhibiting a high correlation. Their analysis also suggested that the intermodular hubs co-express with their interactants in a tissue-restricted manner, and the intramodular hubs co-express with their interactants in almost all tissues. The intermodular hubs also tended to contain cellular signaling domains. Some of those domains were related to oncogenesis. Analyzing gene expression datasets of the breast cancer cohorts revealed that PPINs involving intermodular hubs differed between poor and good prognosis.

Centrality in static networks, which aggregate dynamic networks, may underestimate or overestimate the centralities of certain nodes. To address this issue, Lerman K. et al. extended the concept of α-centrality to dynamic networks [55]. This metric is based on the intuition that in a dynamic network, for a node to influence other nodes over a certain period of time, there must be edges, with or without intermediate nodes, connecting the initial node to affected nodes at different times. For the scientific article citation network, the authors claimed that dynamic centrality provides information that cannot be found with static centrality.

Taylor D. et al. combined snapshots of the centrality matrix from multiple graphs to form a supra-centrality matrix. Using this matrix, they considered the eigenvector, PageRank, and HITS centralities [56]. They referred to these as joint centralities because it assigns centrality to a pair (v,t) for node v and time t. They also introduced the concepts of marginal and conditional centrality.

### 3.4. Path- and Walk-Based Centralities in Dynamic PPINs

In dynamic PPINs, both the presence of interactions and their temporal ordering influence information propagation and connectivity between proteins. As described in Section 2, temporal networks require the consideration of time-respecting paths, in which interactions must occur in a valid chronological sequence. Therefore, to accurately capture communication processes in time-evolving networks, it is essential to extend path- and walk-based centrality measures to dynamic settings. In this section, we review dynamic centrality measures based on paths and walks in temporal PPINs.

Pan R.K. et al. investigated temporal paths in empirical networks of human communication and air transportation, based on the definition of average temporal distances among nodes and an algorithmic implementation [57]. Their analysis showed that the temporal distance correlated with the static distance, but significant differences existed. They also observed that the difference between static and temporal centralities was larger for temporal closeness centrality than for other centrality measures.

Based on paths that exist at any given time, Magnien et al. proposed a temporal extension of the notion of centrality in dynamic networks [58]. Furthermore, they applied this centrality to the dataset and confirmed that the importance of a node could vary significantly, indicating that identifying nodes that are consistently important over time could become meaningless.

To understand the structure of temporal networks, Takaguchi T. et al. defined two centrality measures based on the fastest temporal paths involving temporal nodes. They also showed that these centralities were robust to changes in the time scale [59]. They confirmed that the distribution of these two centrality measures is non-uniform in real-world dynamic networks, and that in some datasets, many temporal nodes with high centrality are located within a narrow time window.

### 3.5. Other Centralities for Dynamic PPINs

In addition to extensions of local topological and path-based centralities, a variety of dynamic centrality measures have been proposed to capture the complex nature of time-evolving networks. As discussed in Section 1 and Section 2, PPINs exhibit heterogeneous dynamics, condition-dependent interactions, and transient connectivity patterns. These characteristics cannot always be fully captured by conventional centrality frameworks.In this section, we introduce representative dynamic centrality measures that go beyond traditional classifications, incorporating temporal heterogeneity, stochastic processes, and communication dynamics.

Various forms of dynamic centrality have been investigated in other areas, but not necessarily in biological applications. Braha D. et al. analyzed centrality using communication records in a large social network and found that the role of nodes changed dramatically from day to day [60]. In particular, they confirmed that local hubs exhibit a power-law distribution over time and do not exhibit a characteristic degree centrality.

To capture rapid topological changes in dynamic PPINs, Kim H. et al. introduced the concept of a “time-ordered graph”, a mathematical model that transforms a dynamic network into a static graph with directed temporal flows [61]. This framework enables conventional graph-theoretical properties, including node centrality measures originally developed for static graphs, to be extended to time-resolved network snapshots. In their study, temporal degree, closeness, and betweenness centralities were defined, demonstrating how static centrality concepts could be extended to dynamic network analysis.

Meligy M. introduced temporal communication centrality to evaluate the communication ability of nodes in social networks [62]. Through a study of percolation in the French cattle trade network, Hoscheit P. et al. confirmed that the dynamic centrality “TempoRank”, based on the random walk introduced by Rocha L.E.C. et al. [63,64], outperformed that defined on a static graph.

To clarify the differences among centrality measures used in PPIN analysis, Table 3 summarizes representative static and dynamic centralities in terms of their required data, analytical tasks, evaluation criteria, biological applications, and methodological limitations.

## 4. Protein Complex Network Construction Using Hypergraphs

PPINs have traditionally been modeled as graphs, in which nodes represent proteins and edges denote pairwise interactions. However, such representations are insufficient for describing protein complexes composed of multiple proteins interacting simultaneously. For example, consider a network consisting of three nodes, each connected to the others by edges. In this representation, it is impossible to distinguish whether the network corresponds to a single protein complex composed of three interacting proteins or to three independent pairwise interactions. Moreover, if these interactions occur at different time points in a dynamic PPIN and are subsequently aggregated into a static representation, they become indistinguishable, all appearing as three nodes connected by three edges.

In recent years, hypergraph-based approaches have attracted increasing attention. Fundamental concepts originally developed for graphs, such as centrality, community detection, and link prediction, have been extended to hypergraphs, demonstrating the utility of hypergraph-based methods in various applications. In the context of protein analysis, a hypergraph can be constructed by representing proteins as nodes and protein complexes as hyperedges. We refer to this representation as a protein complex network (PCN). Applying hypergraph-based analytical methods to PCNs is expected to yield novel biological insights. Several studies have proposed the use of hypergraphs for protein analysis [19,65,66].

Klamt S. et al. reviewed the growing recognition of hypergraphs as a modeling framework for network analysis in cell biology [19]. Their review introduced the concept of hypergraphs, highlighted their differences from conventional graphs, and discussed applications of hypergraph theory in biological network analysis. Battiston F. et al. presented various frameworks for modeling networks beyond pairwise interactions and reviewed their relationships to existing concepts and representations [65]. They also introduced structural measures and models, including bipartite graphs and hypergraphs, for characterizing such systems.

### 4.1. Topological Structure

Several studies have characterized the global topological structure of PCNs by treating them as hypergraphs. Here, the global topological structure refers to the overall patterns and properties of connectivity that emerge when a network is analyzed at the network-wide level.

Ramadan E. et al. modeled the yeast proteome as a hypergraph-based PCN to uncover its underlying structure [67]. They confirmed that the yeast proteome hypergraph exhibits both scale-free and small-world properties. Scale-free networks are characterized by the presence of highly connected nodes known as “hubs”, while small-world networks are those in which most nodes can be reached from any other node in a relatively small number of steps, exemplified by the “six degrees of separation” concept, whereby any two individuals are connected by six or fewer social links. In this study, the authors developed an algorithm to compute the k-core of a hypergraph, a sub-hypergraph consisting of nodes with a degree of at least k, which represents a “sub-complex” in a biological context. This algorithm was used to identify the “core proteome”, defined as the largest core within the PCN. The yeast core proteome was found to contain a high proportion of homologous and essential proteins. These findings likely reflect fundamental biological properties of yeast, including the lethality associated with the deletion of certain proteins and the rapid nature of its biological responses.

Estrada E. et al. presented several examples of networks that are difficult to represent using conventional graph models, such as social and biological networks. To address this limitation, they proposed the concept of “complex hypernetworks” based on hypergraphs [68] and extended network measures such as subgraph centrality, which they had previously introduced [51], as well as clustering coefficients. Subgraph centrality measures the extent to which a node participates in different sub-hypergraphs, while the clustering coefficient quantifies network transitivity based on the ratio of hyper-triangles (closed triplet hyperedges) to paths of length two (open triplet hyperedges). To capture higher-order interactions, hypergraph-based extensions of network measures can be effectively utilized for representation and analysis.

Gallagher S.R. et al. investigated the physical and biological significance of various definitions of clustering coefficients for hypergraphs, including newly proposed variants, in the context of PPIs [69]. They also evaluated the usefulness of these measures for predicting protein complexes in hypergraph representations. Their benchmarking analysis demonstrated that multiple hypergraph clustering coefficients (e.g., hypergeometric, union, geometric, and maximum) outperformed conventional graph-based measures in identifying protein pairs that belonged to the same functional module. However, the authors emphasized that the choice of an appropriate coefficient should be tailored to the specific biological dataset under analysis. They further concluded that the use of a two-node (pairwise) clustering coefficient is more appropriate than that of a one-node (single-node) clustering coefficient for predicting proteins that are common components of protein complexes. This is likely because the pairwise clustering coefficient directly captures co-membership relationships between proteins within complexes.

### 4.2. Centrality Measures in Hypergraphs

Some centrality measures originally defined for graphs can also be extended to hypergraphs. Kapoor K. et al. extended the concept of node degree centrality to hypergraphs, initially focusing on applications in social networks [70]. They also investigated methods for weighting hyperedges and introduced two distinct types of node centrality: strong-tie and weak-tie node degree centrality. Strong ties refer to the repeated occurrence of specific nodes within a limited number of dense hyperedges. In contrast, weak ties are characterized by nodes participating in a diverse set of distinct hyperedges. Their results showed that weak-tie node degree centrality outperformed strong-tie node degree centrality in predicting influence in hypergraph-based social network datasets. This is likely because the nodes that span multiple groups with weak ties act as “bridges”, facilitating the flow of influence between otherwise disconnected communities.

Benson A.R. et al. employed a tensor-based representation as an extension of adjacency matrices for graphs to model uniform hypergraphs [71]. Here, “uniform” refers to hypergraphs in which all hyperedges have the same size, meaning that they are homogeneous. Specifically, they defined three types of eigenvector centrality based on eigenvalues and eigenvectors, whose existence is guaranteed by the Perron–Frobenius theorem extended to tensors. They demonstrated the usefulness of these centrality measures by analyzing hypergraphs derived from n-gram frequencies (sets of n letters or words in text), co-tagging patterns on Stack Exchange, and drug combinations observed in outpatient emergency settings. This tensor-based framework provides a natural way to represent higher-order interactions by directly modeling multi-way relationships, rather than decomposing them into pairwise connections.

Klimm F. et al. demonstrated through various analyses that hypergraphs are more suitable than graph-based models for examining PPIs [72]. They showed that when PCNs, originally represented as hypergraphs, were projected onto pairwise interaction graphs, the proteins identified as “hubs” differed between the two representations. Furthermore, they found that the distribution of local clustering coefficients changed markedly between the hypergraph model and the pairwise interaction graph. Based on these findings, they argued that analytical methods relying solely on pairwise interactions may yield misleading or incomplete results.

TuĞAl I. et al. proposed a centrality measure based on the entropy of nodes and hyperedges in hypergraphs. They argued that entropy is well-suited for identifying influential nodes, as it provides a robust measure of uncertainty [73]. In their study, entropy was calculated using node degree, intersection similarity, and Jaccard similarity, which is an index that captures not only the presence of common elements but also their proportion relative to the total size of the combined sets. These measures were then compared locally. Metrics based on node degree-based and union similarity-based metrics showed higher precision in capturing local centrality, reflecting the strength of a node’s association within its community. In contrast, intersection-based and Jaccard similarity measures served as indicators of global centrality, representing a node’s ability to bridge distinct communities.

Collectively, these studies highlight that centrality in hypergraphs cannot be captured by a single notion but rather depends on the type of higher-order interactions and the structural role of nodes.

### 4.3. Learning and Clustering Methods for Hypergraphs

Several analytical methods developed for graphs have been extended to hypergraphs and are expected to also be applicable to PCNs. Michoel T. et al. extended the Perron–Frobenius theorem to hypergraphs for clustering and community detection [74]. This theorem guarantees that the components of the corresponding principal eigenvector are strictly positive under appropriate conditions, a property that has enabled the development of spectral clustering algorithms for both directed and undirected hypergraphs. The authors demonstrated the potential of this approach for aligning PPINs across multiple species and for detecting functional clusters in cellular signaling pathways.

Lugo-Martinez J. et al. presented a hypergraph-based approach for modeling biological and cellular systems, in which node classification, edge classification including hyperedges, and link prediction problems in graphs and hypergraphs are formulated within a unified framework [75]. These tasks are formulated as node classification problems on (extended, dual) hypergraphs and are treated as semi-supervised learning problems. They also introduced a kernel method for hypergraphs and evaluated its potential for estimating the number of missing and false-positive links in PPINs.

Feng Y. et al. proposed a hypergraph neural network (HGNN) framework for data representation learning [76]. By encoding data structures as hypergraphs, this approach enhances the flexibility of data modeling for complex datasets. Experiments on citation network classification and visual object recognition tasks have shown that HGNN outperforms graph convolutional neural networks and other conventional methods. The framework has also demonstrated superior performance when handling multimodal data. In recent deep learning approaches, learning the structure of graphs or hypergraphs has become a fundamental basis for model development.

Jiang J. et al. pointed out that important latent relationships in the inherent structure are not always directly captured by graphs and hypergraphs [77]. To address this limitation, they developed dynamic hypergraph construction (DHG), a module for dynamically updating hypergraph structures, and hypergraph convolution (HGC), a module for encoding higher-order relationships within the hypergraph framework. By stacking DHG and HGC modules, they proposed a hypergraph neural network framework (DHGNN). Its performance was evaluated on the Cora citation network and a microblog dataset, where it showed competitive or superior performance compared with existing state-of-the-art methods.

Sharma A. proposed an analytical framework based on hypergraph modeling for the analysis of data with higher-order relationships [78]. The author also highlighted the usefulness of employing different hypergraph models depending on the problem, as well as the need for developing inference methods that incorporate temporal information and algorithms for compressing hypergraphs into lower-dimensional latent spaces.

To address scalability challenges in large hypergraphs, Maleki S. proposed “HyperNet”, a multilevel algorithmic framework for representation learning on very large hypergraphs [79]. This framework employs bipartite graph-based representations to efficiently handle complex structures while preserving hypergraph information without information loss. In addition, the author introduced “BiPart”, a fast, parallel, and deterministic hypergraph partitioning algorithm designed for scalable analysis. This algorithm enables efficient processing of large-scale hypergraphs and has potential applications in diverse domains, including integrated circuit design, sparse matrix computations, and large-scale data storage.

In sum, these approaches illustrate a shift from theoretical spectral methods to scalable and learning-based frameworks for analyzing complex hypergraph data.

## 5. Machine Learning and Deep Learning Methods for PPI and PPIN Prediction

As briefly introduced in the previous sections, machine learning and deep learning techniques have been applied to the analysis of PPINs, including hypergraph-based approaches. Here, we summarize recent advances in the application of these methods.

### 5.1. Sequence-Based Frameworks for PPI and PPIN Prediction

In traditional machine learning approaches for PPI prediction, various computational methods have been developed. For example, You Z.H. et al. proposed a computational method called PCA-EELM, which combines principal component analysis (PCA) and ensemble extreme learning machine (E-ELM) to quickly and accurately predict PPIs based solely on the amino acid sequence information of proteins [80]. Similarly, Li Y. et al. developed a computational method that combines the position-specific scoring matrix (PSSM), the MatFLDA feature extraction algorithm, and a random forest (RF) classifier [81]. This method can effectively predict PPIs using only protein amino acid sequences. Furthermore, Karabulut O.C. et al. developed a machine learning model called ML-AdVInfect, which employs a support vector machine (SVM) and integrates predictions from various virus-host PPI prediction tools along with the taxonomic information of the host, to specifically predict whether a given adenovirus can infect a particular host species [82]. Moreover, Coelho E.D. et al. developed a computational model to predict PPIs between humans and oral microbes [83]. They integrated five different PPI prediction techniques and trained a naive Bayes classifier using experimental data from multiple high-quality databases. This model successfully predicted a large number of high-confidence human-oral microbe PPIs, which may play important roles in the development of oral diseases. Their results provide valuable datasets and networks that enhance our understanding of host–microbe interactions in the oral microenvironment and may help identify potential drug targets and biomarkers.

Building on these traditional machine learning techniques, deep learning has emerged as a powerful alternative, offering the ability to automatically learn complex feature representations from raw data, thus reducing the need for manual feature engineering. Zhang F. et al. developed a deep learning-based prediction method named DeepSG2PPI [84]. They first extracted and encoded protein sequence information, local contextual information, and global statistical features derived from protein sequences, and constructed a graph representing the relationships between proteins and GO annotations. Graph embedding techniques were then employed to obtain vector representations of the proteins. DeepSG2PPI combined a two-dimensional convolutional neural network (2D-CNN) with a one-dimensional convolutional neural network (1D-CNN) model and incorporated an attention mechanism to enhance the prediction performance. Tsukiyama S. et al. proposed a deep learning model for predicting human-virus PPIs [85]. The approach uses an adapted Word2Vec model to encode amino acid sequences into vector embeddings, then applies bidirectional LSTM to capture long-range dependencies and contextual patterns in human and viral protein sequences. The LSTM modules enable the model to handle variable-length inputs and produce fixed-length representations, improving the accuracy of interaction prediction. Similarly, Ieremie I. et al. proposed TransformerGO, a deep learning model that predicts PPIs based solely on GO annotations [86]. By embedding GO terms using node2vec and applying a transformer architecture with self-attention, the model captures complex semantic relationships between GO term sets associated with two proteins. Unlike sequence-based approaches, TransformerGO treats GO annotations as unordered sets and uses attention to highlight the most informative terms, achieving high predictive accuracy and interpretability. Sun T. et al. developed a sequence-based deep learning model, utilizing a stacked autoencoder (SAE) to learn complex patterns from protein sequences. Protein sequences were encoded using autocovariance (AC) and conjoint triad (CT) methods, with AC proving more effective for this task [87]. The multi-layer SAE automatically extracted hierarchical and abstract features from the encoded sequences, enabling the model to achieve high accuracy across benchmark and external datasets, and demonstrating strong generalization to other species.

### 5.2. Graph-Based Frameworks for PPIN Prediction

Deep learning techniques also extend into graph-based learning, where PPIs are modeled through complex network structures. GNNs, which generalize deep learning to graph data, have become increasingly crucial for capturing the topological and relational properties of biological networks. For example, Qiu J. et al. developed a network-based method for predicting cancer-specific PPIs using a relational graph convolutional network (R-GCN). The model uses knowledge-based features and cancer-specific data, and considers the type and direction of gene links, such as activation or phosphorylation, to predict PPIs. The R-GCN learns from the structure of the gene network, achieving high accuracy and revealing key cancer hub genes involved in network perturbations [88]. Xiang Z. et al. developed SN-GGAT, a deep learning model for predicting PPIs by representing them as signed networks and improving GAT. They introduced a gating mechanism and adapted the attention mechanism to handle both positive and negative edges, allowing the model to learn from higher-order neighbors and better follow balance theory. SN-GGAT outperformed existing methods on yeast and human PPI datasets, showing that enhanced GAT with gating can improve the prediction accuracy in complex biological networks [89]. Wu J. et al. developed DL-PPI, a deep learning model for predicting PPIs based on protein sequence data. The model uses Inception V3 to extract sequence features and a graph isomorphism network (GIN) to learn global topological relationships from PPINs. DL-PPI achieves strong performance by combining GIN with self-attention and a feature-relational reasoning network (FRN), and generalizes well to unknown proteins [90].

Hypergraph-based models offer a more expressive framework for capturing higher-order biological interactions, enabling the representation of multi-protein complexes beyond simple pairwise links. Recent research has increasingly explored hypergraphs to model complex relationships in PPINs more accurately and comprehensively. Hypergraphs allow edges to connect multiple nodes simultaneously, making them especially suitable for modeling protein complexes and multi-protein interactions that simple pairwise connections cannot fully represent. This extension into hypergraph-based learning and integrating multi-modal biological data has opened new avenues for more accurate and comprehensive PPI prediction. As one of the key advances in this area, Xia S. et al. developed HyperGraphComplex, a deep learning model that predicts protein complexes by integrating protein sequence features with higher-order topological structures from PPINs using hypergraph-based learning [91]. The model employs a hypergraph variational autoencoder (HGVAE), which captures complex relationships among proteins by learning latent feature representations through hypergraph convolution and variational inference. Unlike traditional feature-engineered models, HGVAE enables the model to effectively encode both local and global interactions without manual feature design, leading to improved prediction accuracy and generalization across species. In addition to static hypergraph models, dynamic network structures have also been explored. Gopalakrishnan S. et al. proposed a novel method using directed hypergraphs to analyze PPIs and identify key proteins and enzymes involved in diseases [92]. Their approach represents PPIs as directed hypergraphs, capturing complex multi-protein relationships, and applies hypergraph-based depth-first search and minor hypergraph reduction to simplify the network structure. This method effectively uncovers disease-relevant protein clusters by leveraging higher-order pathway information, outperforming traditional graph algorithms in detecting influential disease-related proteins and enzymes. Focusing on small molecule modulation of PPIs, Zhang Z. et al. proposed HiGPPIM, a hierarchical graph neural network for predicting small molecule protein–protein interaction modulators (PPIMs) [93]. The model uses a two-level graph structure and a hypergraph attention network (HyGAT) to combine atom-level and functional group-level features, capturing complex molecular interactions. HyGAT introduces dual attention mechanisms, linking atoms and functional groups through a hypergraph, allowing for better feature learning and improving prediction performance.

### 5.3. Advanced Computational Frameworks for PPIN

Ye X. et al. developed a stochastic hypergraph model to represent contact relationships between amino acid residues within proteins [94]. The model captures the statistical features of multi-residue interactions and enables the detection of non-random associations in protein families and databases. Tran L. constructed hypergraph models from gene expression datasets to predict gene function by applying three semi-supervised learning methods (un-normalized-, random walk-, and symmetric normalized-hypergraph Laplacian) on hypergraphs [95]. These three models demonstrated that the performance was higher than that based on the un-normalized graph Laplacian, not the hypergraph-based method. Chen J. et al. proposed a hierarchical multi-label learning model “FHML” for predicting the intracellular locations of proteins [96]. In this model, latent concepts, such as codebooks that articulate protein features and their annotations, are extracted by feature space decomposition and label space decomposition. A dual fuzzy hypergraph was used to extract the intrinsic higher-order relations embedded in the feature and label spaces. Chitra U. proposed a hypergraph model of gene interaction networks and validated it with an analysis method using random walks on hypergraphs [97]. The application of the model enabled the ranking of disease genes using the PageRank algorithm. It was demonstrated that the results of the analysis with hypergraphs significantly outperformed those with graphs in the case of monogenic diseases. Feng S. et al. used a new dataset of host transcription responses to pathogenic virus infection, constructing the relationships between genes as a hypergraph model [98]. The hyperedge in their hypergraphs represent the genes perturbed by viral infection. The nodes (or vertices) represent biological samples under specific experimental conditions. The centrality measures on the hypergraph, newly introduced by the authors, were demonstrated to be superior to graph centrality in identifying genes important for complex biological phenomena such as viral infection responses. Surana A. et al. proposed two approaches as a new framework for a hypergraph similarity measure (HSM) to compare hypergraphs [99]. One approach converts hypergraphs into graphs using methods such as clique and star expansion, and then applies graph similarity measures. The other approach defines the similarity measures from tensor-based representations of hypergraphs using tensor algebraic concepts. The advantages and disadvantages of these approaches were investigated, and their performance was evaluated on biological and other datasets. Murgas K.A. et al. analyzed dynamic gene expression data with a hypergraph model and quantified network heterogeneity by Forman–Ricci curvature [100]. They found an increased global curvature of the network in pluripotent stem cells and cancer cells. They also performed pathway analysis on the melanoma dataset and found increased local curvature in several oncogenic pathways and decreased curvature in cancer-suppressive pathways. They also compared the results with graph-based models and gene expression differencing approaches. To represent PPIs and analyze PPIs in diseases, Gopalakrishnan S. et al. used directed hypergraphs as a model [92]. Their goal was to predict proteins related to diseases such as Parkinson’s disease, COVID-19, and diabetes. In particular, they newly introduced the depth-first search algorithm for directed hypergraphs to investigate their properties by searching for the longest pathway. They found that minor hypergraphs (sub-hypergraphs obtained by the contraction of hyperedges and the removal of partial hypergraphs) represent the longest paths, resulting in unimodular hypergraphs. In such cases, unimodular hypergraphs that cluster related proteins and enzymes lead to solutions for developing disease treatments.

Among other emerging computational methods, reinforcement learning (RL) has gained attention as a dynamic and adaptive approach for optimizing protein complex networks (PCNs). Unlike traditional models that rely on static heuristics, RL can simulate biological decision-making processes, discovering optimal protein interactions through iterative learning and feedback. Palukuri M.V. et al. developed an RL-based method to detect protein complexes in PPINs by learning optimal walk strategies, modeling complex detection as a sequential decision task [101]. In this framework, an agent expands subgraphs guided by a value function, which links subgraph density to the probability of forming a true complex. This approach not only improves efficiency and scalability but also enables the exploration of complex structures beyond simple dense subgraphs, with value functions transferable across different network contexts.

Generative models represent another innovative direction in PPI prediction, focusing on the ability to create new interaction hypotheses from learned biological patterns. By modeling the complex distribution of known PPIs, approaches such as variational autoencoders (VAEs) and generative adversarial networks (GANs) can suggest novel PPIs that might not yet be experimentally verified, aiding in the discovery of hidden components within biological networks. Wang J. et al. developed a deep molecular generative framework specifically based on GANs for the de novo design of PPI inhibitors. In this model, GANs are combined with 3D convolutional neural networks to learn from molecular shapes and pharmacophore features, enabling the generation of structurally novel molecules optimized for PPI targeting. The framework efficiently explores chemical space, producing candidate compounds with enhanced drug-likeness and high similarity to known PPI inhibitors [102]. In another application of generative models, Zhang Y. et al. proposed a semi-supervised method using VAE for extracting relationships such as PPIs from biomedical texts. The VAE allows the model to learn from labeled data and large amounts of unlabeled data, improving relation extraction when labeled data are limited. By encoding sentences about PPIs into a latent space and reconstructing them, the VAE helps the classifier better predict PPI relations, showing strong performance, especially in low-resource settings [103]. Table 4 summarizes the key AI-based models applied to PPI prediction.

### 5.4. Practical Considerations for Model Comparison

Taken together, the studies discussed above indicate that recent AI-based approaches for PPI and PPIN prediction differ not only in architecture, but also in the type of biological information they use and the level of interaction they are designed to represent [76,80,81,82,83,84,85,86,87,88,89,90,91,92,93,94,95,96,97,98,99,100,101,102,103,104,105,106,107,108]. Sequence-based models are generally most useful when reliable network topology is unavailable, because they can learn directly from amino acid composition, sequence context, or functional annotations [80,81,82,83,84,85,86,87,104,105]. In contrast, graph neural networks are most informative when the underlying PPIN is sufficiently reliable and biologically meaningful, since their predictive advantage depends on whether neighborhood structure provides a signal beyond pairwise sequence similarity [88,89,90]. Likewise, hypergraph-based methods are particularly relevant when the biological objective is to model multi-protein complexes or other higher-order relationships that cannot be represented faithfully by pairwise edges alone [19,65,76,91,92,106]. Reinforcement learning and generative models further broaden this methodological landscape by supporting sequential search, candidate generation, and exploratory hypothesis formulation, although they should not be regarded as universal replacements for supervised PPI classifiers [101,102,103].

At the same time, the performance of these models is shaped not only by architecture, but also by dataset design and evaluation strategy. In PPI prediction, benchmark results may vary substantially depending on how negative samples are constructed, how class imbalance is handled, whether homologous proteins or protein pairs are rigorously separated between training and test sets, and whether graph-based models are evaluated in transductive or inductive settings [109,110,111,112,113]. These evaluation choices can lead to misleading conclusions. For example, unrealistic negative sampling or poorly controlled class imbalance may make the task artificially easy or produce deceptively high accuracy even when a model mainly predicts negatives [109,110]. Likewise, insufficient separation of homologous proteins or overlapping protein pairs between training and test sets can inflate performance because the model may exploit sequence similarity, repeated interaction patterns, or degree-related bias rather than learn transferable biological determinants of interaction [110,111]. For graph-based methods, evaluation in transductive and inductive settings reflects different levels of generalization difficulty, with performance often declining under inductive settings involving unseen proteins [112,113]. In addition, curated interaction databases are often biased toward well-studied proteins and pathways, which may inflate apparent generalization. Therefore, model families should be compared not solely by predictive accuracy, but also by their biological scope, data requirements, interpretability, and robustness under realistic validation conditions [114,115]. Table 4 provides a structured comparison of representative AI-based frameworks for PPI and PPIN prediction in terms of their typical inputs, major strengths, principal limitations, and appropriate application scenarios.

**Table 4:** Comparison of AI models for PPIs and PPINs prediction.

Model Type	Typical Input	Main Strengths	Limitations/Use	References
Traditional ML	Sequence, structural features	Interpretable, strong baseline	Feature engineering dependent/pairwise prediction	[80,81,82,83]
CNN	Sequence, embedding	Local motif extraction	Limited network context/sequence-based PPI	[84]
RNN/LSTM	Sequence, embedding	Sequential dependency modeling	Harder scaling/sequence-based PPI	[85]
Transformer	Sequence, GO terms, pretrained embeddings	Context-rich representations	Computationally intensive/large-scale inference	[86,104,105]
Autoencoder	Encoded sequence or multimodal features	Compression, latent embedding	Lower interpretability/representation learning	[87,103]
RL	Dynamic PPIN or PCN	Adaptive search	Reward-sensitive/complex detection	[101]
GNN	PPIN graph	Topology-aware learning	Graph-quality dependent/link prediction	[88,89,90]
HGNN	Hypergraph or PCN	Higher-order modeling	Hyperedge-quality dependent/complex prediction	[76,91,92,106,108]
Generative models	Molecule, latent graph, or text features	Candidate generation	Needs external validation/exploratory design	[102,103]

## 6. Case Studies

### 6.1. Case Study 1: AI-Driven Reconstruction of Cancer-Specific PPINs

A representative biologically driven case study is provided by NECARE, a cancer-specific framework developed to capture how oncogenic alterations reshape PPINs in real disease settings [88]. In cancer cells, mutations disrupt PPINs, rather than simply causing dysregulation of individual proteins. For example, mutation-induced alterations in TP53 function not only disrupt its canonical interactions with key regulators such as PTEN and MDM2 [116,117], but also facilitate network rewiring, partly through CDK4-mediated phosphorylation of mutant TP53 [117,118]. This case addresses a clear biological question, namely how cancer-associated mutations disrupt existing protein relationships and create new ones that rewire signaling connectivity. To study this problem, real data from multiple sources were integrated. Cancer PPIs curated from KEGG [119], Reactome [120], and OncoPPI [121] were used as supervised interaction labels. A broader background graph of gene and protein relationships was assembled from STRING [122], KEGG, and HIPPIE [123]. These network data were combined with OPA2Vec [124] ontology-based features and TCGA-derived expression and mutation profiles so that the final input reflected both interaction topology and cancer-relevant molecular context. On this basis, a relational graph convolutional network was constructed to explicitly model both the type and direction of biological relationships. This design is particularly relevant in cancer signaling because network edges do not represent a single uniform relation. Instead, they include activation, inhibition, expression-linked associations, and binding relationships, all of which may influence whether a cancer-context PPI is likely to occur [88].

The framework was used not only to classify isolated protein pairs but also to reconstruct a perturbed cancer interactome and examine the broader consequences of network rewiring [88]. NECARE first predicted cancer-specific PPIs. It was then applied to map interactome perturbations across the cancer network, showing that genes on average lost existing edges but gained even more new ones. This finding supports the view that the cancer interactome is actively reprogrammed rather than simply damaged. In addition, a total of 1293 cancer hub genes enriched for interaction perturbations were identified and grouped into genes mainly associated with gained links, lost links, or both. These hub classes were non-random. Type 1 hub genes were enriched in major oncogenic pathways such as MAPK, PI3K–Akt, and Wnt signaling, and hub gene mutation burdens were associated with prognosis across 32 cancer types. In this way, the study moved beyond simple link prediction and connected network-level predictions to biologically and clinically meaningful outputs, including signaling rewiring, pathway enrichment, and disease-relevant hub prioritization.

Model performance was evaluated quantitatively through cross-validation and comparison with alternative sequence-based and network-based methods [88]. NECARE achieved an F1 score of 91 ± 2%, a Matthews correlation coefficient (MCC) of 0.84 ± 0.03, and an area under the curve (AUC) of 0.97 in the reported test evaluation, and it also maintained strong performance on an independent dataset. A reliability index was further introduced to show that higher-confidence predictions were associated with higher precision, which increased the usefulness of the output for downstream biological analysis. Accordingly, performance assessment in this case was not limited to a single metric, but included classification accuracy, discrimination ability, benchmarking against competing methods, and confidence calibration.

Most importantly, NECARE incorporated direct experimental validation in a biologically meaningful signaling context [88]. Predicted crosstalk around WNT3 from the Wnt pathway and SHC2 from the Ras pathway was selected for validation, and these predictions were tested in LN229 glioblastoma cells using co-immunoprecipitation. Among the 20 tested interactions, 18 were confirmed experimentally, corresponding to 90 percent validation accuracy. This makes the study especially valuable as a case study because it links a biologically relevant signaling setting to a concrete validation workflow. Real pathway data were used to define the network setting, the model predicted cancer-specific interactions, prediction strength was quantified, and targeted wet-lab validation was performed on selected high-confidence candidates.

### 6.2. Case Study 2: Hypergraph-Based Learning for Protein Complex Discovery

A central challenge in protein complex analysis is that the biological target is not merely a pairwise interaction, but a higher-order assembly composed of multiple coordinated subunits. Conventional graph-based representations are often limited in this setting because they reduce complex organization to collections of binary edges, which may fail to preserve the structure of true multi-protein assemblies. This problem becomes especially important when complexes are sparse, partially observed, or difficult to distinguish from noisy local network modules. HyperGraphComplex was developed in response to this higher-order complex prediction problem, with the goal of identifying biologically meaningful protein complexes more effectively than topology-only or pairwise frameworks [91].

To address this limitation, HyperGraphComplex reformulated the protein complex prediction task using hypergraph modeling [91]. Real biological data from multiple sources were integrated into this framework. Curated yeast protein complex benchmarks compiled in AdaPPI [125] were used as positive training references, an independent Complex Portal dataset [126] was used for testing, PPINs from DIP [127], BioGRID [12], and Mann-PPI [128] provided the interaction backbone, and UniProt [129] protein sequences were encoded using the conjoint triad method. The pairwise PPIN was then converted into a hypergraph by treating clique-derived subnetworks as hyperedges, so that candidate complexes could be represented as higher-order units rather than simple sets of binary interactions. On this basis, a hypergraph VAE was used to learn latent representations that jointly capture sequence information and higher-order network topology, and these embeddings were then passed to a deep neural network classifier for complex prediction. In this way, the method linked the biological problem of complex organization to a modeling framework that is structurally more appropriate for multi-protein assemblies.

The effectiveness of the framework was demonstrated at multiple levels. At the predictive level, HyperGraphComplex outperformed existing methods such as ClusterOne [130], AdaPPI, and Node2vec-RF [131] across standard evaluation metrics including the F1-score, precision, recall, and complex-wise accuracy. At the mechanistic level, ablation experiments showed that performance declined when the hypergraph framework was replaced by a conventional graph VAE, and also declined when either sequence features or PPI topology was removed, indicating that both higher-order topology and sequence-derived information contributed to the final performance. At the biological level, predicted complexes showed stronger GO semantic similarity, GO enrichment, and expression concordance than random pseudo-complexes, suggesting that they retained functional coherence similar to known complexes. At the external support level, several predicted complexes were validated by independent experimental studies, including CK2-Gag1, SAMMco6, Thp3-Csn12-Sem1, CROP, and Kar4-Vir1-Dyn2 related complexes [128,132,133,134,135]. Finally, three predicted complexes were further supported by high-confidence AlphaFold-Multimer [136] structures, providing an orthogonal structural layer of validation [91].

### 6.3. Case Study 3: CNN-Based Analysis of Protein Localization Dynamics and Its Implications for PPINs

At each stage of the cell cycle, including G1, S, G2, and M phases, post-translational modifications such as phosphorylation and dephosphorylation are known to regulate protein activity, interactions, and subcellular localization [137,138]. Because the progression of the cell cycle is relatively well-defined, it has been widely used as a model system for studying time-dependent changes in protein interactions [138]. However, directly observing dynamic processes such as the formation and dissociation of interactions or transitions of protein complexes remains challenging. This is primarily because time-series proteomics and phospho-proteomics mainly measure protein abundance and modification states, making it difficult to capture PPINs and their spatial co-localization directly and comprehensively.

To address this limitation, Litsios et al. first constructed yeast strains that expressed endogenous proteins fused with a green fluorescent protein (GFP) tag, as well as three types of red and far-red fluorescent proteins [139]. This approach was taken to investigate changes in protein localization and concentration during the cell cycle. They then developed a method for identifying yeast cells and determining protein localization using live-cell imaging. This approach enabled the simultaneous identification of cell cycle stages and subcellular compartments. They also combined a supervised CNN framework called CycleNet, which was trained on cell cycle images, with a model called DeepLoc [140] for the automated classification of subcellular protein localization. This allowed them to extract dynamic changes in protein localization at single-cell resolution. Furthermore, protein abundance was quantified based on fluorescence intensity from the acquired images. Thus, this study extracted localized and highly accurate cell cycle information from time-lapse images and constructed a comprehensive spatiotemporal proteome map that serves as the basis for dynamic PPIN reconstruction.

The study used this approach to demonstrate that a large number of proteins exhibit periodic changes in subcellular localization and/or abundance in coordination with cell cycle progression. These findings suggest that PPINs are not only governed by changes in binding relationships, but also by spatial constraints determining whether proteins can coexist within the same cellular compartment. Therefore, this study highlights the potential of image-based deep learning approaches to provide spatially resolved, dynamic information that can inform the reconstruction of time-dependent PPINs. However, this study did not directly predict or verify the interaction itself, but rather should be positioned as a foundational data layer that provides the spatial and temporal constraints necessary for dynamic PPIN reconstruction. In the future, verifying the candidate interactions and complex transition hypotheses derived from this study is expected to yield a more detailed understanding. This could be achieved using independent experimental methods such as mass spectrometry imaging, proximity labeling, and immunoprecipitation with cells synchronized to each stage of the cell cycle.

## 7. Conclusions and Future Directions

PPINs serve as essential maps for understanding how proteins work together to carry out cellular functions. Although traditional static models have provided critical insights into pairwise protein relationships, they are inherently limited in describing the dynamic, cooperative, and context-dependent nature of real biological systems. In this review, we discussed the field’s progression from simple graph representations to more advanced models, including dynamic networks and hypergraph-based approaches. Each of these developments has significantly improved the accuracy and biological relevance of PPI modeling [19,21,22]. Dynamic PPINs incorporate time-series data such as gene expression profiles, allowing researchers to analyze how interaction patterns shift under different conditions or stimuli. These models have enabled the identification of transient modules and context-specific protein complexes [25,26,27,28,29,30,31]. However, limitations remain, including sparse time points in public datasets, interpolation challenges, and the difficulty of capturing interactions that are conditionally expressed or short-lived [37,38,141].

In parallel, centrality measures have evolved to reflect the temporal and structural complexity of biological networks. Traditional metrics such as degree, betweenness, and eigenvector centrality have been complemented by temporal centralities that account for how a protein’s role can vary across different time points or conditions [43,45,46,47,48,49,50,51,55,56,57,58,59]. These metrics provide powerful tools to prioritize candidate hub proteins and understand network dynamics in health and disease [53,54]. Hypergraph modeling offers a fundamentally different perspective by allowing multi-protein interactions to be represented as single entities. This approach captures higher-order structures that are essential in processes such as transcriptional regulation, signal transduction, and chromatin remodeling [19,65,68,69]. It also resolves ambiguities that arise in pairwise-only models, where multiple configurations may appear indistinguishable despite having different biological implications [19,67]. The integration of artificial intelligence has further propelled this field forward. GNNs, HGNNs, and generative models such as VAEs and GANs provide flexible frameworks for learning from complex biological data [76,84,85,87,88,89,91,101,102,103,104,105,106]. When combined with domain-specific information such as GO annotations, protein sequences, and localization data, these models can accurately predict unknown interactions and uncover latent biological structures [86,87,90,104].

Nevertheless, computational predictions must be grounded in biological reality. Experimental validation using methods such as co-immunoprecipitation, proximity labeling, or perturbation assays (Table 1) should be performed [142,143]. The iterative feedback loop between modeling and experimentation will be crucial to ensure the reliability and utility of future discoveries. Building upon these foundations, the next phase of research is expected to embrace even more expressive, data-integrative, and predictive modeling strategies. One promising direction is the application of generative graph models such as variational graph autoencoders [144]. These models are capable of inferring missing or intermediate interaction states that cannot be directly observed in experiments. For example, they may help reconstruct transient interaction complexes during processes such as viral reactivation. Once predicted, such intermediate states can be embedded into hypergraph-based protein complex networks to support community detection, centrality analysis, and the exploration of latent regulatory structures [91].

Dynamic hypergraph-based learning algorithms also present exciting opportunities. Models such as dynamic hypergraph convolutional networks and temporal motif detectors are expected to enhance our ability to represent how protein complexes evolve over time [76,106,145]. Furthermore, the integration of multi-modal biological data, including expression levels, post-translational modifications, and spatial distributions, will improve the contextual fidelity of these models [92,107]. Reinforcement learning further adds an adaptive layer by simulating biological interventions. For example, agents trained to modify hyperedges could mimic gene knockdowns or drug treatments, providing an in silico platform for hypothesis generation and therapeutic screening [101,146,147]. Ultimately, any prediction must be experimentally validated, reinforcing the importance of coupling computational innovation with biochemical evidence [142,143].

Taken together, the convergence of dynamic modeling, hypergraph theory, and machine learning points to a transformative era in PPI research. This integrated perspective will enable researchers to unravel the complexity of PPIs with greater precision, leading to deeper biological insights and more effective strategies for diagnosing and treating diseases.

## Figures and Tables

**Table 1:** Overview of representative experimental methods for PPI detection and their principles.

Experimental Methods for PPI	Experimental Principle	Data Scale	Representative Methods
Yeast two-hybrid	In yeast cells, the bait protein is expressed as a fusion with a DNA-binding domain. The prey proteins are often expressed as a library of fusion proteins with a transcriptional activation domain. The PPI between the bait and prey is assessed by reporter gene activation, which enables selection based on growth or survival of the cell.	High-throughput	Yeast two-hybrid
Complex reconstitution	Recombinant proteins are primarily used as bait to evaluate in vitro interactions with either specific recombinant proteins or endogenous cellular proteins present in cell lysates. When recombinant proteins are used for both the bait and the prey, direct physical interactions can be assessed.	Small-scale/target-specific	GST pull-down/gel-shift/surface plasmon resonance (SPR)
Co-crystal structure	Direct PPI at the atomic level can be demonstrated by X-ray crystallography, nuclear magnetic resonance (NMR), or electron microscopy (EM). These methods are also capable of detecting protein complexes composed of three or more components. In recent years, Cryo-EM has become widely used, as it enables structural analysis without the need for protein or complex crystallization.	Target-specific	NMR, EM, Cryo-EM
Affinity capture-Western blotting	Bait proteins are affinity-captured from cell extracts using specific antibodies or epitope tags. Interactions are evaluated by detecting co-precipitated endogenous proteins with specific antibodies or co-expressed, epitope-tagged proteins via Western blotting. These approaches may also detect indirect PPIs.	Small-scale/Target-specific	Immuno-precipitation/tag-specific pull-down
Resonance energy transfer	Interactions are assessed by fluorescence resonance energy transfer (FRET) between donor–acceptor pairs (e.g., CFP- and YFP-tagged proteins) that occurs when the proteins are in close proximity. Various fluorescent protein derivatives have also been developed for this purpose. Bioluminescence resonance energy transfer (BRET), based on luciferase-generated luminescence, is also widely used.	Small-scale/target-specific	FRET/BRET
Affinity capture-MS	Bait proteins are affinity-captured from cell extracts using specific antibodies or epitope tags. Interactions are evaluated by detecting co-precipitated endogenous proteins with specific antibodies or co-expressed, epitope-tagged proteins via Western blotting. Recombinant proteins can also be used as bait to capture interacting proteins from cell extracts.	High-throughput	Immuno-precipitation mass spectrometry (IP-MS)
Proximity label-MS	Enzyme (e.g., BioID/APEX) fusion proteins are used as bait to selectively label proximal prey proteins with molecules such as biotin. These labeled proteins are subsequently affinity-captured and identified by mass spectrometry.	High-throughput	BioID/APEX

**Table 2:** Comparison of static and dynamic PPIN modeling approaches.

Methods	Data	Task	Evaluation	Application	Limitations
Graphs (static PPIN)	PPI data (BioGRID, STRING), structural information, interaction networks	Network analysis, centrality analysis, module detection	Topological metrics (degree, clustering), correlation (PCC)	Basic PPIN analysis, hub protein identification, functional inference	Cannot capture temporal changes; cannot explicitly represent higher-order (multi-protein) interactions; static assumption
Dynamic graphs (dynamic PPIN)	Time-series gene expression data, PPI + GO, time-dependent data	Dynamic network construction, complex detection, network interpolation and prediction	Accuracy (complex detection accuracy), temporal consistency, correlation	Disease state comparison, time-dependent PPI analysis, functional module analysis	Data dependency (resolution and noise of time-series data); interpolation issues (estimation between snapshots); high computational cost; inability to handle unknown interactions

**Table 3:** Summary of representative centrality measures in PPINs.

Methods	Data	Task	Evaluation	Application	Limitations
Degree centrality (DC)	Graph structure (PPIN)	Detection of hub proteins	Correlation with essentiality, etc.	Identification of important proteins	Local-only measure; sensitive to noisy or incomplete PPINs.
Eigenvector centrality (EC)	Adjacency matrix (global structure)	Evaluation of influence	Eigenvalue/eigenvector analysis	Detection of highly influential proteins	Hub-biased; sensitive to heterogeneity and missing data; computationally costly in large networks.
Betweenness centrality (BC)	Shortest-path information	Evaluation of bottlenecks and information flow	Path-based metric	Inter-module connectivity; signaling pathway analysis	Assumes shortest-path flow; misses redundant/probabilistic pathways; computationally expensive.
Closeness centrality (CC)	Shortest-path distances	Evaluation of network accessibility	Inverse of average shortest-path distance	Evaluation of information diffusion efficiency	Distance-dependent; ignores local motifs; unstable in fragmented or incomplete PPINs.
Bottleneck centrality (BNC)	Shortest-path tree	Detection of structural bottlenecks	Number of downstream nodes	Identification of important connector nodes	Definition- and threshold-sensitive; varies with network structure and conditions.
Subgraph centrality (SC)	Adjacency matrix (all walks)	Evaluation of motif participation	Weighted sum of closed walks	Detection of essential proteins	Computationally expensive; difficult to interpret biologically; hard to scale.
Dynamic centrality	Time-series networks	Evaluation of time-dependent importance	Temporal paths; temporal distances	Dynamic PPIN analysis; disease analysis	Requires high-resolution time-series data; sensitive to noise, temporal resolution, and snapshot construction.

## Data Availability

No new data were created or analyzed in this study. Data sharing is not applicable to this article.

## Abbreviations

The following abbreviations are used in this manuscript:

PPINs	Protein–protein interaction networks
PPIs	Protein–protein interactions
SWI/SNF	SWItch/sucrose non-fermentable
TS-OCD	Time smooth overlapping complex detection model
TAP	Tandem affinity purification
GO	Gene ontology
DPCT	Dynamic method to detect protein complexes from the TAP-Aware weighted PPI network
Regime-SSM	Regime-state space model
ARTIVA	Auto Regressive TIme VArying
SGD	*Saccharomyces* Genome Database
TINCD	Two-layer integrated complex detection
KGs	Knowledge graphs
GNNs	Graph neural networks
DGNN	Dynamic graph neural network
DC	Degree centrality
PCC	Pearson’s correlation coefficient
ECC	Edge clustering coefficient
WDC	Weighted degree centrality
EC	Eigenvector centrality
HITS	Hyperlink-induced topic search
CC	Closeness centrality
BC	Betweenness centrality
BNC	Bottleneck centrality
SC	Subgraph centrality
PCA	Principal component analysis
PCN	Protein complex network
DL	Deep learning
CNNs	Convolutional neural networks
RNNs	Recurrent neural networks
LSTM	Long short-term memory
HGNNs	Hypergraph neural networks
GCNs	Graph convolutional networks
RL	Reinforcement learning
DHG	Dynamic hypergraph construction
HGC	Hypergraph convolution
E-ELM	Ensemble extreme learning machine
PSSM	Position-specific scoring matrix
RFs	Random forests
SVM	Support vector machine
1D-CNN	One-dimensional convolutional neural network
2D-CNN	Two-dimensional convolutional neural network
AC	Auto covariance
CT	Conjoint triad
R-GCN	Relational graph convolutional network
GIN	Graph isomorphism network
FRN	Feature-relational reasoning network
HGVAE	Hypergraph variational autoencoder
PPIMs	Protein–protein interaction modulators
GAT	Graph attention network
HyGAT	Hypergraph attention network
HSM	Hypergraph similarity measure
VAEs	Variational autoencoders
GANs	Generative adversarial networks
MCC	Matthews correlation coefficient
AUC	Area under the curve
GFP	Green fluorescent protein
